# Low Variation in the Polymorphic *Clock* Gene Poly-Q Region Despite Population Genetic Structure across Barn Swallow (*Hirundo rustica*) Populations

**DOI:** 10.1371/journal.pone.0028843

**Published:** 2011-12-21

**Authors:** Roi Dor, Irby J. Lovette, Rebecca J. Safran, Shawn M. Billerman, Gernot H. Huber, Yoni Vortman, Arnon Lotem, Andrew McGowan, Matthew R. Evans, Caren B. Cooper, David W. Winkler

**Affiliations:** 1 Fuller Evolutionary Biology Program, Cornell Lab of Ornithology, Cornell University, Ithaca, New York, United States of America; 2 Department of Ecology and Evolutionary Biology, Cornell University, Ithaca, New York, United States of America; 3 Department of Ecology and Evolutionary Biology, University of Colorado, Boulder, Colorado, United States of America; 4 Department of Zoology and Physiology, University of Wyoming, Laramie, Wyoming, United States of America; 5 Department of Zoology, Tel Aviv University, Tel Aviv, Israel; 6 School of Biosciences, University of Exeter, Exeter, United Kingdom; 7 Cornell Lab of Ornithology, Cornell University, Ithaca, New York, United States of America; Smithsonian Institution National Zoological Park, United States of America

## Abstract

Recent studies of several species have reported a latitudinal cline in the circadian clock gene, *Clock*, which influences rhythms in both physiology and behavior. Latitudinal variation in this gene may hence reflect local adaptation to seasonal variation. In some bird populations, there is also an among-individual association between *Clock* poly-Q genotype and clutch initiation date and incubation period. We examined *Clock* poly-Q allele variation in the Barn Swallow (*Hirundo rustica*), a species with a cosmopolitan geographic distribution and considerable variation in life-history traits that may be influenced by the circadian clock. We genotyped Barn Swallows from five populations (from three subspecies) and compared variation at the *Clock* locus to that at microsatellite loci and mitochondrial DNA (mtDNA). We found very low variation in the *Clock* poly-Q region, as >96% of individuals were homozygous, and the two other alleles at this locus were globally rare. Genetic differentiation based on the *Clock* poly-Q locus was not correlated with genetic differentiation based on either microsatellite loci or mtDNA sequences. Our results show that high diversity in *Clock* poly-Q is not general across avian species. The low *Clock* variation in the background of heterogeneity in microsatellite and mtDNA loci in Barn Swallows may be an outcome of stabilizing selection on the *Clock* locus.

## Introduction

By providing the opportunity to explore the association between genetic variation and adaptive phenotypic differentiation, candidate genes contribute to our understanding of the genetic basis of traits that are of ecological importance [1], [2]. The circadian clock, which generates endogenous ∼24 hr biochemical, physiological and behavioral rhythms, has been fully characterized at the molecular genetics level (reviewed by [3]). The circadian clock gene, *Clock*, has been sequenced from hundreds of taxa, and in vertebrates it codes for a protein (CLOCK) that serves as a transcription factor (as a heterodimer with BMAL1) in the core circadian clock oscillator [4]–[6]. The *Clock* gene is highly conserved among vertebrates throughout most of its sequence; however, its C-terminal region contains a variable poly-glutamine (poly-Q) motif with variability in the number of glutamine repeats both among and within species [7]–[9]. This region is known to influence the transcription activating potential of the protein, and therefore it can affect both behavior and physiology related to circadian rhythms [10], [11].

*Clock* poly-Q variation provides an opportunity to examine its association with life-history traits that may be related to circadian clock. Studies on various taxa have reported an association between *Clock* genotype and breeding latitude [9], [12], [13]. In birds, latitudinal variation in *Clock* genotypes have been detected in the Blue Tit (*Cyanistes caeruleus*) but not for the Bluethroat (*Luscinia svecica*) [9] or *Tachycineta* swallows [14], therefore the generality of this pattern in birds and its possible relationship with adaptive phenotypic differentiation remains unclear. Additional within-population analyses showed an association between *Clock* genotype and breeding phenology (clutch initiation date and incubation duration) in a Blue Tit population [15] but not in a sympatric Great Tit (*Parus major*) population, in which *Clock* poly-Q variation was low [16]. Thus, further comparisons are required to assess patterns of association between *Clock* variation and life-history traits.

The Barn Swallow (*Hirundo rustica*) is one of the world's most widely distributed passerines, with a broad geographic distribution throughout most of the northern hemisphere. The six described subspecies [17]–[20] exhibit substantial geographic variation in morphometric characteristics (such as tail streamer length and ventral coloration) and in life-history traits such as migration patterns and time of breeding [19], [21], [22]. This species' wide geographic distribution combined with life-history variation make it a good candidate in which to examine the generality of the association between *Clock* poly-Q and life-history traits related to the timing of seasonal reproductive behavior.

In this study we examined *Clock* poly-Q allelic length variation in five breeding populations of Barn Swallows (from three subspecies). These populations differ in several life-history traits related to breeding latitude, migratory behavior and breeding phenology (Table 1). We sampled three populations of the New World subspecies, *H. r. erythrogaster*, from New York, California and from a recently established breeding population in Argentina [23], and two populations from the Old World: a non-migratory population from Israel (*H. r. transitiva*) and a population from the United Kingdom (*H. r. rustica*). The Old World and New World clades are divergent in mtDNA with ∼1.6% sequence difference [24], [25]. Our main objectives were to (a) characterize the *Clock* poly-Q region in Barn Swallows, (b) examine genetic variation among pairs of five populations sampled from three subspecies in relation to differences in their life history traits and (c) compare genetic differentiation at the *Clock* locus to that of presumably neutral loci (mitochondrial DNA haplotypes and microsatellite loci). Comparisons among the *Clock* locus and loci that are selectively neutral provide an important background to examine whether the *Clock* locus is under selection or exhibits patterns of neutral evolution.

**Table 1:** Characteristics of the Barn Swallow populations used in this study with sample sizes for the genetic markers used in this study.

Population	Subspecies	Breeding latitude	Migratory behavior	Time of breeding	Breeding cycles^1^	# of samples analyzed for		
						*Clock*	ND2	Microsatellites
Israel	*transitiva*	32°55′N	No^2^	Feb–Jun	2 (3)	49	31	178
United Kingdom	*rustica*	50°42′N	Yes	Apr–Sep	2	49	30	62
New York	*erythrogaster*	42°30′N	Yes	May–Aug	2	49	31	41
California	*erythrogaster*	38°N	Yes	May–Jul	2	31	19	23
Argentina	*erythrogaster*	38°30′S	Yes	Oct–Feb	2 (3)	52	63	63

1 Typical number of breeding cycles with maximum breeding cycles in parentheses.

2 Barn Swallows from Israel only move locally outside the breeding season.

## Methods

### Study populations and sample collection

We studied five breeding populations of Barn Swallows representing three of the six described subspecies. In each of these breeding populations we collected blood samples from adults during the breeding season and stored them in lysis buffer until DNA was extracted. Population characteristics and sample sizes used in this study are presented in Table 1.

### Analysis of Clock poly-Q alleles

**Figure 1:**
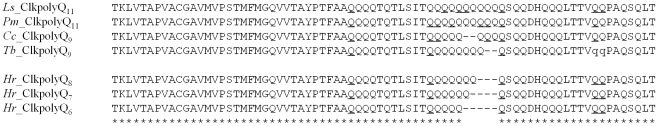
Amino acid alignment of avian CLOCK polyQ alleles. Alignment includes sequences from Barn Swallows (*Hirundo rustica*; *Hr*) together with published Blue Tit (*Cyanistes caeruleus*; *Cc*), Great Tit (*Parus Major*; *Pm*), Bluethroat (*Luscinia svecica*; *Ls*) and Tree Swallow (*Tachycineta bicolor*; *Tb*). For each sequence the species name and number of Clock poly-Q repeats are shown. The predicted protein sequences of Barn Swallow *Clock* poly-Q repeats only differ in the number of CAG codon (coded by Q) repeats (The first and last glutamine (Q) amino acids in the poly-Q repeat were coded by CAA codons). Q residues coded by CAA are underlined and lower-case Qs are within-population polymorphic site encoded by either CAA or CAG. Asterisk indicates identical amino acids in the poly-Q flanking region between the all sequences.

Genomic DNA was extracted from blood using the E-Z 96 Tissue DNA kit (Omega Bio-Tek, Norcross, GA) or using the DNAeasy blood Extraction kit (Qiagen, Valencia, CA). In order to examine variability in Barn Swallow *Clock* poly-Q region and to verify the genetic sequence, we first amplified this region (corresponding to human *Clock* gene exon 20 [26]) from 3–5 individuals from each of the Barn Swallow populations using the sequencing primers developed by [9]. 10 µL PCR amplifications included 1 µL undiluted DNA, 10 µM Tris-HCl , 50 µM KCl, 4 mM MgCl2, 0.25 mM of each nucleotide, 0.25 mM from each primer, and 0.025 U jumpstart Taq polymerase (Sigma-Aldrich, St. Louis,Missouri). PCR amplification conditions were: initial denaturation at 95°C for 4 min 30 s; 30 cycles of denaturating at 95°C for 1 min, annealing at 64°C for 1 min, and extension at 72°C for 2 min; and a final extension at 72°C for 4 min 30 s. PCR products were purified using Exonuclease and Shrimp Alkaline Phosphatase enzymatic reactions (United States Biochemical, Cleveland, OH). Purified products were cycle sequenced in both directions using amplification primers and ABI BigDye Terminator chemistry. Sequencing products were cleaned using Sephadex columns and electrophoresed in an ABI 3730 Automated DNA Analyzer (Applied Biosystems, Foster City, CA). We aligned forward and reverse strands for each specimen and checked them using Sequencher 4.7 (Gene Codes Corp., Ann Arbor, MI). The amplified sequence generated for all Barn Swallows matched the expected sequence for this gene for birds. In total, we sequenced the *Clock* poly-Q region for 17 Barn Swallows (GenBank accession numbers JN986726-JN986747). All Barn Swallow sequences were identical to each other and differed only in the number of poly-Q repeats (Fig. 1). The first and last glutamine amino acids in the poly-Q repeat were coded by CAA codons, whereas the middle codons were exclusively CAG. Thus, the only variation was in the number of CAG codons.

We then screened 49 adults from Israel, 49 from the United Kingdom, 49 from New York, 31 from California and 52 from Argentina for length polymorphism in the *Clock* poly-Q region using the genotyping primer set developed by [9] in which the forward primer was labeled at the 5′ end with 6-FAM fluorescent dye. The PCR protocol was similar to the one used for sequencing (see above). PCR products were genotyped on an ABI 3100 Genetic Analyzer (Applied Biosystems) with GeneScan-500 LIZ (Applied Biosystems) as the molecular size standard. Allele sizes were estimated using Genemapper v3.7 (Applied Biosystems) together with control samples with known repeat numbers determined by sequencing. We were able to successfully genotype all Barn Swallow samples (N = 230).

### Analysis of mitochondrial sequences and microsatellite loci

Mitochondrial and microsatellite loci for the Israeli and UK populations were analyzed as part of a population genetics study which focus on the Old World samples [27], while loci for the three New World populations were analyzed as part of a population genetics study which focus on the New world samples [28]. The same individuals were used across all markers in all populations. Sample sizes for the *Clock* marker and the two neutral markers (microsatellites and mtDNA) are not equal due to sample and data availability. However, similar analyses with a subset of individuals that were analyzed for all three markers (for all five populations) generated similar results. Therefore, we report here only results using all available samples.

We amplified and sequenced the mitochondrial protein-coding gene nicotinamide adenine dinucleotide dehydrogenase subunit 2 (ND2) for a subset of each population sample (Table 1). We used primers METb and TRPc [29]. 10 µL PCR amplifications included 1 µL undiluted DNA, 10 µM Tris-HCl , 50 µM KCl, 3–4 mM MgCl_2_, 0.25 mM of each nucleotide, 0.25 µM from each primer, and 0.025 U jumpstart Taq polymerase (Sigma-Aldrich). PCR amplification conditions were as follows: initial denaturation at 95°C for 4 min 30 s; 30–35 cycles of denaturating at 95°C for 1 min, annealing at 54°C or 62°C for 1 min, and extension at 72°C for 2 min; and a final extension at 72°C for 4 min 30 s. PCR products were purified using Exonuclease and Shrimp Alkaline Phosphatase enzymatic reactions (United States Biochemical). Purified reactions were cycle sequenced in both directions using amplification primers and ABI BigDye Terminator. Sequencing products were cleaned using Sephadex columns and processed with an ABI 3730 Automated DNA Analyzer (Applied Biosystems). We aligned forward and reverse strands for each specimen and checked them using Sequencher 4.7 (Gene Codes Corp.). All sequence data were deposited in GenBank (Accession Nos. JN642465-JN642524 and HQ333550-HQ333663).

A total of six nuclear microsatellite loci previously developed for *H. rustica* or other birds were analyzed for all populations: Escµ6 [30], Ltr6 [31], POCC6 [32], Hir11, Hir19 and Hir20 [33]. Forward primers were labeled at the 5′ end with fluorescent tags (PET, 6-FAM, VIC or NED; Applied Biosystems). Individual PCR amplifications were combined into multiplex mixes to decrease the number of reactions needed per individual. 10 µL PCR amplifications included 10–100 ng of genomic DNA, 10 µM Tris-HCl , 50 µM KCl, 1.5–3.25 mM MgCl_2_, 0.25 mM of each nucleotide, 0.12–0.24 µM from each primer, and 0.025 U jumpstart Taq polymerase (Sigma-Aldrich). PCR amplification conditions were as following: initial denaturation at 95°C for 5 min; 35 cycles of denaturating at 95°C for 30 s, annealing at 50°C or 58°C for 30 s (specific per multiplex PCR mix), and extension at 72°C for 30 s; and a final extension at 72°C for 30 min. PCR products were genotyped on an ABI 3100 Genetic Analyzer (Applied Biosystems) with GeneScan-500 LIZ (Applied Biosystems) as molecular size standard. Allele sizes were estimated using Genemapper v3.7 (Applied Biosystems) and verified and amended by eye to ensure standardized data collection.

### Statistical analysis

Observed and expected heterozygosities for the *Clock* poly-Q region for each population were calculated using ARLEQUIN v3.11 [34]. We tested for departures from Hardy-Weinberg equilibrium (HWE) per locus and population and for deviation from linkage equilibrium (LD) for all microsatellite loci combinations using GENEPOP v4 [35], [36] with parameters of 10,000 dememorization, 10,000 batches, and 10,000 iterations. We estimated overall genetic differentiation between populations using *F_ST_* values in ARLEQUIN v3.11 [34]. We applied a sequential Bonferroni correction [37] to these test results.

We used Pearson correlation matrix in order to examine the relationship between pairwise genetic differentiation values (*F_ST_*) between populations estimated from the various genetic markers.

Statistical analysis was done using Statistica 7.0 (Statsoft, Inc., Tulsa, OK). Results are presented as mean ± SE unless stated otherwise.

### Ethics Statement

This study was conducted under the permits of Israel Nature Reserve Authority (permit numbers 28234-2007 and 31345-2008), UK Home Office (project license number 30/2740), Dirección de Administración de Areas Protegidas y Conservación de la Biodiversidad, Provincia Buenos Aires, Argentina (collecting permit number 6607) and the U.S. Fish and Wildlife Service (Federal Bird Banding Permit number 20576).

## Results

### Clock poly-Q variation in Barn Swallows

We genotyped a total of 230 Barn Swallows from five populations representing three subspecies. Overall, we found only three different length-variant alleles, ClkpolyQ_6, 7, 8_, corresponding to 6–8 poly-Q repeats coded by CAG codons (Table 2). However, one allele, ClkpolyQ_7_, accounted for 98% of the overall allelic diversity and 96.5% of individuals were homozygous for this allele. We found all three alleles in only one population (Israel), whereas all individuals from one population (Argentina) were homozygous to the ClkpolyQ_7_ allele. Mean observed heterozygosity (*H* = 0.030) was correspondingly low and ranged from 0.000 for the Argentina population to 0.082 for the Israeli population.

**Table 2:** *Clock* poly-Q allele frequencies, number of alleles (*k*), mean allele size (with standard errors) and observed heterozygosities (*H*) for the five Barn Swallow populations used in this study.

Population	Subspecies	N	*k*	Mean allele size (s.e.)	Allele proportion			*H*
					Q_6_	Q_7_	Q_8_	
Israel	*transitiva*	49	3	6.96 (0.03)	0.051	0.939	0.010	0.082
United Kingdom	*rustica*	49	2	6.99 (0.01)	0.010	0.990	0.000	0.020
New York	*erythrogaster*	49	2	7.01 (0.01)	0.000	0.990	0.010	0.020
California	*erythrogaster*	31	2	7.02 (0.02)	0.000	0.984	0.016	0.032
Argentina	*erythrogaster*	52	1	7.00 (0.00)	0.000	1.000	0.000	0.000
**Total**		230	3	6.99 (0.01)	0.013	0.980	0.007	0.030

Allele frequencies did not deviate from Hardy-Weinberg equilibrium for all populations (*P*>0.05).

Owing to the low variation in *Clock* poly-Q alleles, we could test for deviation from HWE only in the Israeli population, which did not show deviation from HWE (*P* = 0.150).

### Population frequencies of microsatellite alleles

We genotyped Barn Swallows from the five populations at six polymorphic microsatellite loci. Mean number of alleles for all loci was 11.83 (±4.50 SD) and ranged from 5.40 for Ltr6 to for 14.80 for Escu6 (minimum number of alleles was 4 for Ltr6 in California and maximum was 21 for Hir20 in Israel). Most of the populations did not deviate from HWE; however we detected deviation from HWE for the Argentina population in Hir11 and for the New York population in Escu6 and Hir19 (after sequential Bonferroni correction). We found no evidence for significant LD for any pair of microsatellite loci.

### Molecular characterization of mitochondrial haplotypes

We aligned most of the ND2 gene (1023 bp) for 174 individuals from the five populations and detected 45 unique haplotypes: 21 for the Old World populations and 24 for the New World populations, which represented two discrete clades (minimum of 13 nucleotide changes between them). Out of the 21 Old World haplotypes, two common haplotypes were shared by individuals from the Israeli and UK populations. Three New World haplotypes were shared by individuals from all three New World populations, while two haplotypes were shared by individuals from the New York and Argentina populations.

### Genetic differentiation

We examined genetic differentiation between all pairs of populations based on the different genetic markers. Mean genetic differentiation based on the *Clock* poly-Q locus (*F_ST_* = 0.0095±0.005) was much lower (and not significant) compared to genetic differentiation based on six microsatellite loci (*F_ST_* = 0.038±0.005, all statistically significant) or on mitochondrial haplotypes (*F_ST_* = 0.578±0.115, all statistically significant). Analysis of genetic differentiation for each of the microsatellite loci separately showed that only Hir20 (*F_ST_* = 0.0082±0.003) had similarly low values to the *Clock* poly-Q locus and the other five loci had higher *F_ST_* values (range: 0.021–0.078).

**Figure 2:**
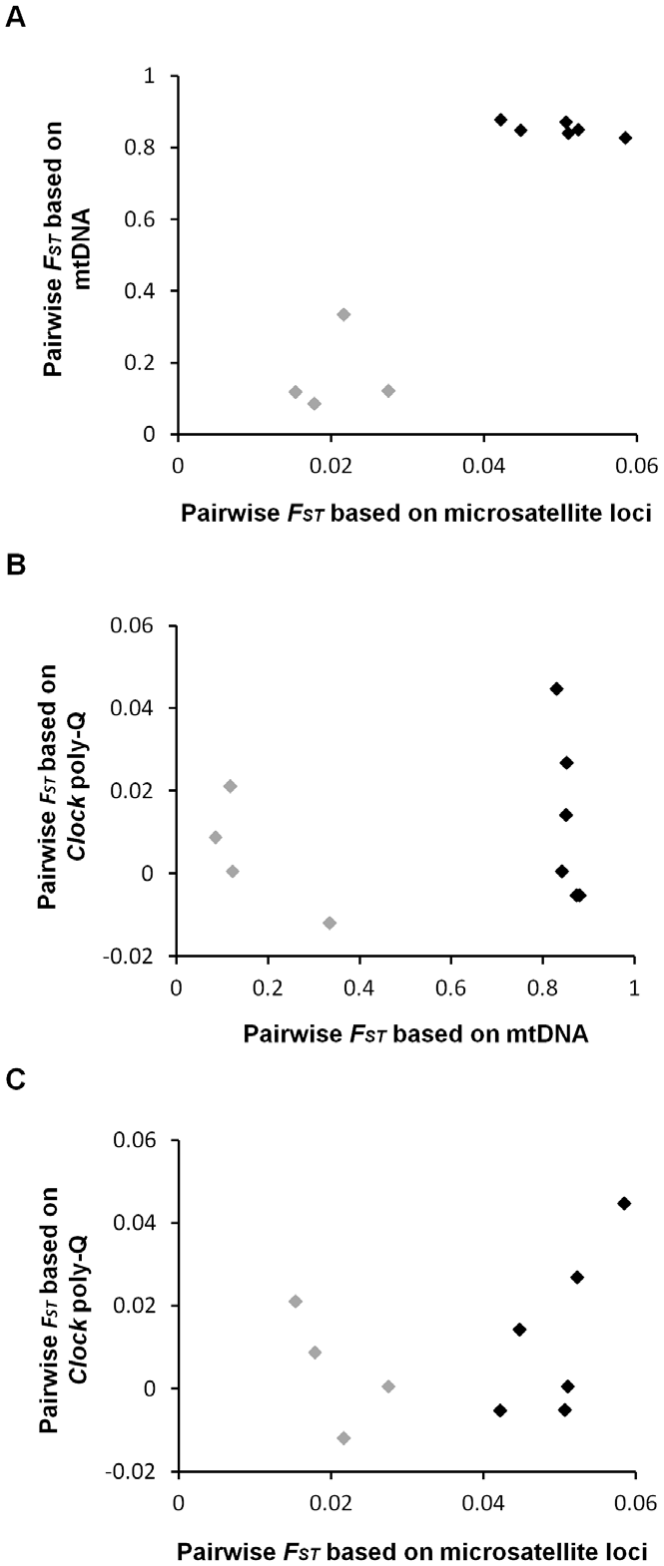
Between-populations pairwise genetic differentiation values (*F_ST_*) comparisons. (A) Comparisons between mitochondrial haplotypes and microsatellite loci, (B) between *Clock* poly-Q locus and mitochondrial haplotypes and (C) between *Clock* poly-Q locus and microsatellite loci. Points in black represent comparisons between the *erythrogaster* subspecies to either the *rustica* or *transitiva* subspecies, while points in grey represent comparison within the *erythrogaster* subspecies and between the *rustica* and *transitiva* subspecies (which are mitochondrially intermixed).

Pairwise genetic differentiation values based on mitochondrial haplotypes and microsatellite loci were highly correlated (Fig. 2A; N = 10, *r^2^* = 0.858, *t* = 6.691, *P* = 0.0001), while similar comparisons between *Clock* poly-Q locus pairwise *F_ST_* values and either mitochondrial haplotypes (Fig. 2B; N = 10, *r^2^* = 0.020, *t* = 0.407, *P* = 0.694) or microsatellite loci (Fig. 2C; N = 10, *r^2^* = 0.110, *t* = 0.995, *P* = 0.349) pairwise *F_ST_* values were not correlated. Separate analyses for each of the microsatellite loci showed that pairwise *F_ST_* values based on these loci were positively correlated with mitochondrial haplotype pairwise *F_ST_* values (range of Pearson correlation coefficient: *r* = 0.237–0.864), although only correlations for Escu6, Ltr6 and Hir11 were significant. Parallel correlations of pairwise *F_ST_* values based on these microsatellite loci to *Clock* poly-Q pairwise *F_ST_* values were not significant and lower than the correlations between pairwise *F_ST_* values of these microsatellite loci and mitochondrial haplotypes (range of Pearson correlation coefficient: *r* = −0.177–0.734), except for Hir19 which showed a significant correlation with the *Clock* poly-Q pairwise *F_ST_* values.

The low variation in *Clock* poly-Q in Barn Swallows precluded additional analysis of the statistical association between *Clock* poly-Q variation and life history traits among the Barn Swallow populations or subspecies.

## Discussion

Variation within the circadian clock core oscillator genes is generally low, but the *Clock* gene poly-Q region shows a unique polymorphism both among and within bird species. This polymorphism is associated with both breeding latitude and breeding phenology in Blue Tits, suggesting that this locus might be involved in local adaptation to seasonal environments, possibly through the response to photoperiod [9], [15]. However, this association has not been detected in several other bird species [9], [16], leaving the question regarding the generality of this relationship unresolved.

Barn Swallows demonstrate a great amount of variation in morphological and life-history traits throughout the Holarctic, including trait diversity that might be related to circadian rhythms such as variation in breeding latitude, time of breeding and migratory patterns. In this study we surveyed five populations of Barn Swallows in order to examine *Clock* poly-Q variation in the species and compare it among Barn Swallow populations that exhibit variation in life-history traits. We identified three *Clock* poly-Q length variants in Barn Swallows, however the variation was extremely low with one allele (Q_7_ – corresponding to 7 poly-Q repeats) accounting for 98% of the total alleles (and two other very rare alleles – Q_6_ and Q_8_), and a mean observed heterozygosity of only 0.030 (range: *H* = 0.000–0.082). This variation is much lower than similar values reported for the Blue Tit (9 alleles; Q_9_-Q_17_; *H* = 0.489), Bluethroat (9 alleles; Q_10_-Q_16_; *H* = 0.213) [9] or even a low-variability Great Tit population (5 alleles; Q_11_-Q_15_; *H* = 0.077) [16] and five species of *Tachycineta* swallows (2–4 alleles; Q_5_-Q_9_; *H* = 0.047–0.472) [14]. This low variation in *Clock* poly-Q in Barn Swallows was also reflected in the genetic differentiation analysis that showed no differentiation among populations based on this locus, while similar analyses based on mtDNA haplotypes or microsatellite loci showed significant and correlated differentiation between these populations (Fig. 2).

Comparison of population genetic differentiation based on the *Clock* poly-Q locus and presumably neutral loci (mtDNA haplotypes and microsatellite loci) showed contrasting findings. While neutral loci reflected, as expected, greater divergence between the Old World and New World clades and less structure within these clades [25], [27] genetic differentiation based on Clock poly-Q locus did not follow such a pattern (Fig. 2). This low variation in the *Clock* poly-Q locus precluded us from associating it with any of the populations' life-history traits (breeding latitude, time of breeding, migration). Still, the sole non-migratory population (*H. r. transitiva* from Israel) showed the highest genetic variation (Table 2). Genetic variation in the non-migratory Blue Tit was also higher compared to the migratory Bluethroat [9]. Yet, a non-migratory Great Tit population showed lower variation than both [16]. Therefore, we cannot assume any general relationship between migratory patterns and genetic variation in the *Clock* poly-Q locus based on the available data.

Why is *Clock* poly-Q variation in Barn Swallows so low compared to other bird species? *Clock* poly-Q allele size in Barn Swallows was shorter compared to those of the other species reported (Q_9–17_ in Blue tit, Q_11–15_ in Great Tit and Q_10–16_ in Bluethroat). Longer tandem repeats are more likely to be more polymorphic, therefore the lower variation observed for Barn Swallows may be related to their relatively shorter *Clock* poly-Q repeats. However, despite long *Clock* poly-Q repeats in a Great Tit population (Q_11–15_), polymorphism was relatively low [16]. Liedvogel and Sheldon [16] suggested that this might be due to the fact that CAG-repeat variation in this locus is tightly regulated and that selection in Great Tit acts in favor of the *Clock* poly-Q_14_ allele. *Clock* poly-Q variation in Barn Swallows in based on CAG-repeats as well, which may be also tightly regulated. However, CAG-repeat polymorphism exists also in the much more polymorphic Blue Tit and Bluethroat [9]; therefore it is less likely to assume that CAG-repeats will be more regulated in one species than the other.

Selection for a specific allele could explain the low variation observed in Barn Swallows (and Great Tits). It is possible that the Q_7_ allele is under positive selection in Barn Swallows (similarly to the Q_14_ allele in Great Tits). However, Johnsen et al. [9] have suggested that the fact that selection has not stabilized the *Clock* poly CAG repeat at a single length in several species is at least suggestive that the variation is itself selectively advantageous. Our results demonstrate homogeneity in the locus in a background of heterogeneity of presumably selectively neutral loci. This scenario suggests that the *Clock* poly-Q variation does reflect the demographic processes among the Barn Swallow population, and is more expected from a selection operating on the *Clock* locus to maintain the low variation while neutral processes, such as genetic drift, may be contribute to the variation in unrelated microsatellite and mtDNA loci [38].

Alternatively, this low variation in the *Clock* poly-Q locus in Barn Swallows might represent a holdover from an ancestral population that itself had low variation in the *Clock* locus. Examination of the *Clock* poly-Q locus in a few individuals of other swallow species in the genus *Hirundo* (*H. angolensis*, *H. aethiopica* and *H. albigularis*) has shown that they are also homozygotes for the Q_7_ allele (Dor R., unpublished data). In addition, the Barn Swallow population from Argentina, which showed no polymorphism in the *Clock* poly-Q locus, was established only in the 1980's, most likely from North American populations [23], [25], [28], thus in this population at least low variation in *Clock* poly-Q may stem from a founder effect. However, even in this newly founded population, presumably selectively neutral loci (mtDNA haplotypes and microsatellite loci) displayed much more variation than the *Clock* poly-Q locus. We cannot exclude the possibility that these presumably selectively neutral loci were already more variable in the ancestral population. However, it is more likely that the *Clock* poly-Q locus in Barn Swallows is not entirely neutral and that some type of selection is operating to reduce variation in the poly-Q region.

The timing of the central clock can be adjusted by several environmental cues. Photoperiod cues have been studied extensively; however the effects of non-photic cues on reproduction have been demonstrated as well [39]. These non-photic cues may include social cues, food and temperature (reviewed in [39]). The mechanisms that enable these non-photic cues to influence circadian rhythms are unknown, however they most likely utilize different genetic pathways. Species and populations may differ in the primacy of each of these cues as the most important cue to adjust reproductive phenology, depending on their ecology. Thus, we might expect genetic differences in species or population reflective of the cues used to adjust their circadian rhythm. For example, if the *Clock* gene is associated with photoperiod, then the nature of selection for this gene (e.g., balancing or positive) could vary depending on whether individuals in that species or population had increased reproductive success relying on non-photic or photoperiod cues. Social cues (associated with mate choice preferences) are very important in determining reproductive success in Barn Swallows [40]–[43], thus it is possible that the lack of *Clock* gene variation reflects social cues primacy over photoperiod cues in this species. Examination of this hypothesis warrants future work.
